# Scoring Targets of Transcription in Bacteria Rather than Focusing on Individual Binding Sites

**DOI:** 10.3389/fmicb.2017.02314

**Published:** 2017-11-22

**Authors:** Marko Djordjevic, Magdalena Djordjevic, Evgeny Zdobnov

**Affiliations:** ^1^Institute of Physiology and Biochemistry, Faculty of Biology, University of Belgrade, Belgrade, Serbia; ^2^Institute of Physics Belgrade, University of Belgrade, Belgrade, Serbia; ^3^Swiss Institute of Bioinformatics and Department of Genetic Medicine and Development, University of Geneva, Geneva, Switzerland

**Keywords:** direct target gene predictions, transcription factor binding site predictions, transcription regulation, position specific weight matrices, transcription targets, transcription start starts, sigma70, bacterial gene expression regulation

## Abstract

Reliable identification of targets of bacterial regulators is necessary to understand bacterial gene expression regulation. These targets are commonly predicted by searching for high-scoring binding sites in the upstream genomic regions, which typically leads to a large number of false positives. In contrast to the common approach, here we propose a novel concept, where overrepresentation of the scoring distribution that corresponds to the entire searched region is assessed, as opposed to predicting individual binding sites. We explore two implementations of this concept, based on Kolmogorov–Smirnov (KS) and Anderson–Darling (AD) tests, which both provide straightforward *P*-value estimates for predicted targets. This approach is implemented for pleiotropic bacterial regulators, including σ^70^ (bacterial housekeeping σ factor) target predictions, which is a classical bioinformatics problem characterized by low specificity. We show that KS based approach is both faster and more accurate, departing from the current paradigm of AD being slower, but more accurate. Moreover, KS approach leads to a significant increase in the search accuracy compared to the standard approach, while at the same time straightforwardly assigning well established *P*-values to each potential target. Consequently, the new KS based method proposed here, which assigns *P*-values to fixed length upstream regions, provides a fast and accurate approach for predicting bacterial transcription targets.

## Introduction

Identifying targets of transcription regulators (transcription targets), such as genes that are directly regulated by a given transcription factor, or transcribed by a certain σ factor, is a crucial step toward understanding bacterial gene expression regulation. Such knowledge is in turn crucial for both biotechnology applications and fundamental understanding of how bacteria respond to changing environment (e.g., during host pathogen interactions).

The task of identifying transcription targets is typically exhibited by starting from either: (i) large scale *in vivo* binding experiments such as ChIP-Seq (Wade et al., 2007; Park, 2009), (ii) large scale *in vitro* binding data, such as high-throughput SELEX (Roulet et al., 2002; Jagannathan et al., 2006) and protein binding microarrays (PBM) (Bulyk, 2006; Newburger and Bulyk, 2009), and (iii) smaller scale experiments, such as SELEX, primer extension (for σ factors) and DNA footprinting (Green et al., 1989; Tuerk and Gold, 1990), which are typically assembled in databases such as TRANSFAC (Wingender, 2008), JASPAR (Mathelier et al., 2016), or RegulonDB (Gama-Castro et al., 2008). From these binding experiments, specificity of a given transcription factor (TF) is then extracted through some of the numerous methods that have been developed for this purpose. Those methods can be based on either information theory considerations (Stormo, 2000; Bulyk, 2004; Favorov et al., 2005; Ozoline and Deev, 2006; Levitsky et al., 2014; Korostelev et al., 2016), or on biophysical models (Stormo and Fields, 1998; Djordjevic et al., 2003; Djordjevic and Sengupta, 2006; Stormo and Zhao, 2010; Vilar, 2010; Djordjevic, 2013; Vilar and Saiz, 2013; Locke and Morozov, 2015), but in either case the inferred DNA binding specificity is represented in a form of a matrix, often called position specific weight matrix (PSWM). Note that, in the case of biophysics based approaches, these PSWMs in fact correspond to the so-called energy matrix (Djordjevic et al., 2003; Stormo and Zhao, 2010). These methods, up to now, have been shown to be able to extract the binding specificity with a reasonable accuracy, particularly when the data are coming from (controlled) high-throughput *in vitro* experiments (Bulyk, 2004, 2006; Djordjevic and Sengupta, 2006).

Once PSWMs are inferred, in prokaryotes they are used to scan genomic regions upstream of potential targets (e.g., the upstream intergenic regions), to find putative direct regulatory targets (Kim and Ren, 2006). These putative targets are next typically compared with the results of high-throughput experiments, such as DNA microarray data, or crosschecked with results of *in vivo* binding experiments (e.g., with the locations of binding peaks from ChIP-Seq experiments). This crosschecking may provide comprehensive information on the underlying regulatory mechanism, e.g., to what extent binding of the regulator under the given experimental conditions matches with the putative list of the genomic regions to which it is expected to bind. Such information is particularly useful when the binding specificity is inferred from *in vitro* binding studies, and is then crosschecked with independent experiments coming from *in vivo* binding measurements (Kim and Ren, 2006; Stormo and Zhao, 2010).

Despite the importance of accurately predicting direct targets for a given regulator, the bulk of the research efforts concentrate on more accurately inferring PSWM. On the other hand, a typical procedure for identifying putative direct targets in bacteria is rather simple, and involves scanning the upstream genomic regions by the inferred PSWM (Kim and Ren, 2006; Wade et al., 2007; Stormo and Zhao, 2010; de Jong et al., 2012). The sites with maximal PSWM scores are then identified, and those above certain thresholds are classified as putative targets. This procedure, however, often results in low search accuracy, in particular, in a very large number of false positives (Robison et al., 1998; Stormo, 2000). In eukaryotes, methods that predict clusters of transcription factor binding sites (TFBS) are also used, in addition to predicting individual TFBS. However, to successfully apply these methods, one often has to know which TFs functionally interact (Hannenhalli, 2008). Also, a recent evaluation shows that the clustering methods lead to lower accuracy compared to individual TFBS predictions (Jayaram et al., 2016). The major reason behind the apparent low accuracy in the search of direct target genes is that individual high-scoring binding sites can easily appear by random chance in a sufficiently long genomic sequence, leading to so called non-sites (Kim and Ren, 2006). While this problem may be, to some extent, alleviated by negative selection acting on these non-sites, this negative selection is likely small. Furthermore, another problem, accurately assigning statistical significance to the targets predicted in such approach is also not well explored. That is, the maximal scoring sites are located in the tale of the weight matrix score distribution, and accurately calculating this tale requires doing an inverse Laplace transform of the corresponding partition function, which, in itself, is an ill-resolved numerical problem (Hertz and Stormo, 1999). Consequently, putative targets above certain threshold are typically reported without assigning statistical significance to the corresponding hits.

To address the problem of accurate transcription target predictions, we here develop a new concept which is based on the following hypothesis. We propose that, rather than identifying individual sites with high weight matrix scores, a better measure is assessing enrichment of the high scoring sites over a certain background in the entire region that is searched. This proposal then does not depend on individual high-scoring sites (which can easily emerge by random), but instead on comparing the weight matrix score distribution for the entire searched region with a certain background distribution. Note that this automatically accounts for the random occurrence of high-scoring binding sites, since such random occurrences (non-sites) would also appear in the background distribution. Moreover, this hypothesis directly couples with elegant statistical methods that allow determining statistical significance of a difference between the two distributions, such as Kolmogorov–Smirnov (KS) or Anderson–Darling (AD) tests. Therefore, these statistical tests also allow straightforwardly assigning a well-established statistical significance to the predicted direct targets, which also addresses the other major deficiency of the usual approach discussed above. Consequently, in contrast to the previous approaches, we will here develop a method which is based on assigning P values to fixed length upstream regions (e.g., the upstream intergenic regions in bacteria), rather than picking up only the best scoring PSWM matches (or their clusters).

However, significant questions emerge with regard to our proposed novel concept:

(i) Can search based on this hypothesis indeed identify direct targets with high accuracy? Does the classification threshold, based on statistical significance assigned through this approach, lead to high prediction accuracy?
(ii) What is the appropriate background (null distribution)?
(iii) What statistical method is more optimal to implement in the problem, Kolmogorov–Smirnov, Anderson–Darling, or perhaps a combination (hybrid) of these two approaches?

In this proof-of-the-concept paper, we will explore this new method by predicting direct targets for bacterial pleiotropic regulators (σ^70^, CRP, FNR), which present a classical (currently unresolved) bioinformatics problem characterized by low prediction specificity. On the other hand, accurately predicting transcription targets of bacterial regulators is crucial for understanding bacterial gene expression regulation. Considering bacterial regulators also allows a more straightforward interpretation of the obtained results, as complicating issues such as chromatin state/accessibility (Forties et al., 2011; Chen and Bundschuh, 2014; Chereji and Morozov, 2014) that are present in eukaryotes are largely absent here.

## Results and Discussion

### Overrepresentation of PSWM Scoring Distributions

**FIGURE 1:**
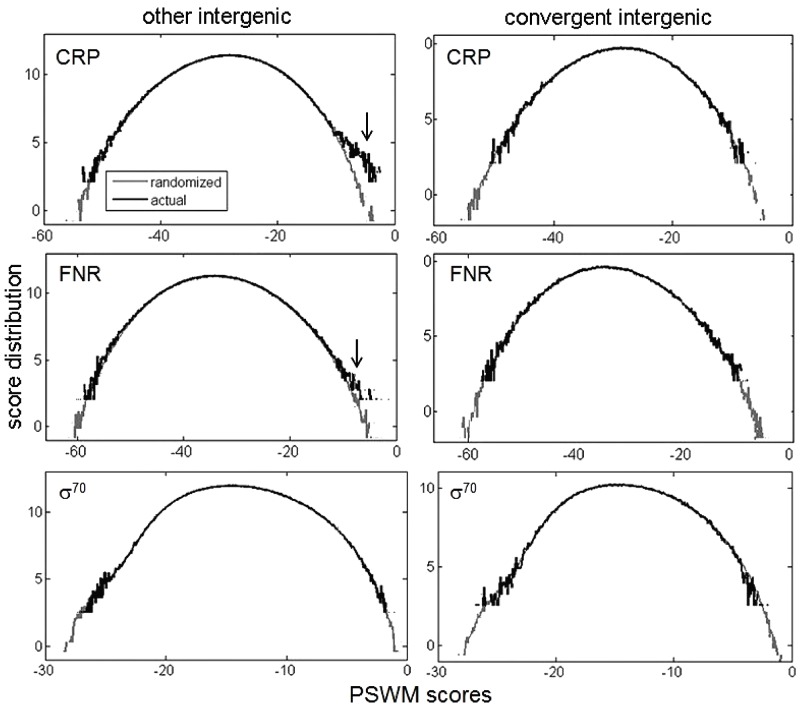
The score distributions for CRP and σ^70^ transcription regulators. The **(upper, middle, lower)** correspond to CRP, FNR, and σ^70^, respectively. The left panels correspond to the other intergenic regions (where functional binding is expected to appear), while the right panels correspond to the convergent intergenic regions (where functional binding is not expected to appear). Other intergenic regions are located upstream of at least one of the adjacent genes, while convergent intergenic regions are located downstream of both of the adjacent genes (by intergenic region we consider the entire sequence between the two adjacent genes). In each figure, the actual and the randomized PSWM distributions are shown in black and gray, respectively. Note that the higher binding scores (closer to zero), correspond to stronger predicted binders. The overrepresentation in the other intergenic regions for CRP and FNR is indicated by arrows.

We start by exploring the basic concept behind our hypothesis that the distribution of PSWM scores is overrepresented in the regions where binding of transcription regulators is expected, and that the overrepresentation is absent in the regions where they do not bind. This concept is illustrated by the upper panel of **Figure 1**, where binding of a pleiotropic *Escherichia coli* transcription factor CRP (also known as CAP) to the convergent intergenic regions, and to the rest of the intergenic regions (here called the “other intergenic regions”), is assessed. Note that the convergent intergenic regions are located downstream of both of the adjacent genes, while the other intergenic regions are located upstream of at least one of the adjacent genes. Therefore, there should be no CRP binding sites in the convergent intergenic regions, while CRP binding sites should be located in a subset of the other intergenic regions, which are upstream of its regulatory targets. Accordingly, in the upper left panel of **Figure 1**, we observe a significant overrepresentation of CRP PSWM scores in the other intergenic regions, while such overrepresentation is absent in the convergent intergenic regions. Note that, in **Figure 1**, the background distribution corresponds to randomized intergenic regions, with the sequences randomized so as to preserve trinucleotide frequencies. We obtain similar results (the middle panels) for another *E. coli* pleiotropic transcription factor (FNR), i.e., we also observe an overrepresentation in the other intergenic regions (though now smaller compared to CRP), and an absence of overrepresentation in the convergent intergenic regions.

On the other hand, a more complex case is presented in the lower panels of **Figure 1**. Here, binding of the *E. coli* σ^70^ factors to the other intergenic (the left panel) and the convergent intergenic (the right panel) regions is assessed. Note that bacterial σ factors ensure transcription initiation (i.e., provide signal for transcription start sites), and different σ factors are related with transcription exhibited under different conditions in a bacterium (Paget and Helmann, 2003; Feklístov et al., 2014). In particular, σ^70^ is the housekeeping σ factor in *E. coli*, which is associated with transcribing a large number of bacterial genes under normal conditions (therefore having a large regulon). We observe an absence of overrepresentation in both the other and the convergent intergenic regions, in fact a small underrepresentation in the high scoring tail for the convergent intergenic regions can be observed. The absence of the overrepresentation is likely a consequence of significant negative selection on σ^70^ non-sites, as a subset of the other intergenic regions (from which transcription of the downstream genes is directed) has to be enriched with σ^70^ binding sites.

σ^70^ binding, in which no global overrepresentation is observed, evidently corresponds to a more complex case of the regulatory target recognition. Consequently, in the results below, we will first concentrate on σ^70^, to demonstrate utility of the method even in a more complicated scenario. In addition, prediction of σ factor binding sites, and their corresponding direct targets (i.e., genes that they transcribe) is a classical (unresolved) bioinformatics problem that is considered notoriously hard (Stormo, 2000; Towsey et al., 2008; Purtov et al., 2014), but one that is crucial for understanding bacterial transcription. Predictions of σ^70^ targets are moreover important since RNA-seq experiments (which can map transcription start-sites) are still rare in bacteria, and a number of transcription start sites are active under non-standard conditions, which likely differ from those used in the experiments (Feklístov et al., 2014). Therefore, an additional motivation is to investigate whether our approach can lead to reasonable predictions for such a difficult problem. We will then come back to analyzing two other *E. coli* pleiotropic regulators (CRP and FNR), which display the more standard/expected binding score distributions.

### Kolmogorov–Smirnov Based Approach

**FIGURE 2:**
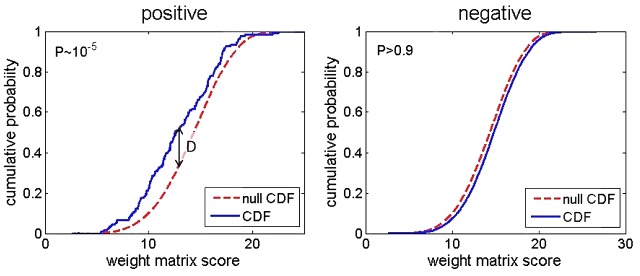
Cumulative distribution functions corresponding to PSWM scores for the upstream genomic regions (the solid blue curve), and the background distribution (the dashed red curve) are shown in the figure. In the **(left)**, an intergenic region that is strongly enriched by σ^70^ binding sites, and that would be reported as a direct target in the search, is shown. In the **(right)**, an extreme case of the upstream intergenic region, which is depleted of σ^70^ binding sites, is shown. *P*-values from KS are indicated in both panels.

The main idea behind the new approach is to observe an overrepresentation of PSWM score distribution for the entire upstream genomic region of interest, with respect to a chosen background (null) distribution. We then need to provide a measure of the difference between the two scoring distributions (corresponding to the upstream genomic regions, and the background distribution), as well as a measure of statistical significance for this difference. Assessing this difference can be directly implemented through Kolmogorov–Smirnov (KS) test, which is illustrated in **Figure 2**.

In the left panel, an example of an upstream intergenic region, which is clearly enriched by σ^70^ binding sites, is shown. The solid curve corresponds to the cumulative distribution function (CDF), corresponding to PSWM scores of this intergenic region. Note that the usual KS measure of the difference between the two distributions (which we here denote as *D* score) is indicated in the figure. With respect to the *D* score, note that we here use the one-sided KS test, i.e., we impose the condition that CDF of the upstream genomic regions has to be above the background distribution CDF, which is the condition that corresponds to overrepresentation – i.e., the case of significant underrepresentation being reported as a hit is excluded. KS test also directly provides the *P*-value corresponding to this *D* score, which in turn allows assessing statistical significance of the potential target. On the other hand, the right panel presents an example where the upstream intergenic region is depleted of σ^70^ binding sites. In this case, CDF of PSWM scores corresponding to this depleted intergenic region is actually below the background distribution, so that the gene downstream of this intergenic region is clearly not reported as a direct target of σ^70^ (*D* score is very close to zero in this case). Note that CDF of the upstream intergenic region does not have to be below the background CDF (as happens in the extreme case shown in the right panel), to be excluded as a hit. That is, all hits with small *D* values, which are statistically non-significant, are not reported as putative targets.

### Enrichment of *D* Scores

**FIGURE 3:**
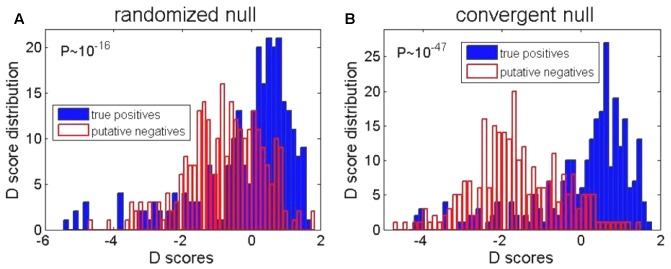
Enrichment of *D* scores for two background distributions. The **(A,B)** represent, respectively, the background distributions that correspond to the randomized and the convergent intergenic regions. The open red and the solid blue histograms, respectively, correspond to the *D* score distributions for the positives (the upstream intergenic regions with experimentally detected binding sites) and the putative negatives (the genomic regions deep inside *E. coli* ORF, where functional σ^70^ binding does not appear). The difference between the blue and the red distributions is assessed by the *P*-value, indicated in each panel.

To implement the KS based method, the choice of the background (null) distribution becomes important. This is actually already indicated in **Figure 1**, where we have seen that, due to the negative selection, the distribution corresponding to the randomized regions may not overlap with the distribution in the regions where no binding happens. We here test two choices of the background distributions: (A) the distribution corresponding to the randomized regions, where the intergenic regions are randomized, and their corresponding PSWM scoring distribution is used as the background, (B) genomic regions where functional binding is not expected, for which we use the convergent intergenic regions, as explained in **Figure 1**. Note that these two choices correspond, respectively, to the left (the randomized regions) and the right (the convergent intergenic regions) panel shown in **Figure 3**.

For each of these two choices of the background distributions, the *D* score distribution is calculated in the following two cases: (i) the red histogram, which corresponds to positives (i.e., the upstream intergenic regions, which are experimentally known to contain σ^70^ binding sites); (ii) the blue histogram: which corresponds to putative negatives, i.e., the genomic regions where σ^70^ binding should *not* appear. Specifically, we here use genomic sequences deep inside ORF (coding sequences), where we expect no initiation of transcription (i.e., no functional σ^70^ binding). We here mark such regions as putative negatives.

We see a significant enrichment of *D* scores in the true positive vs. putative negative regions, for both choices of the background distributions (i.e., for both **Figures 3A,B**). However, we see that the enrichment is clearly much higher when the background distribution corresponds to the convergent intergenic regions, as clearly indicated by the *P*-values in the **Figures 3A,B**. The most likely reason is that the randomized regions do not capture (possibly significant) negative selection that acts on σ^70^ binding sites. That is, the functional binding, which one needs to detect, comes on the ‘top’ of possibly a large number of non-sites that are ‘deleted’ by the negative selection. Consequently, in the further analysis, we will use the background distribution which corresponds to the convergent intergenic regions.

### ROC Curves and Comparison with Anderson–Darling Test

**FIGURE 4:**
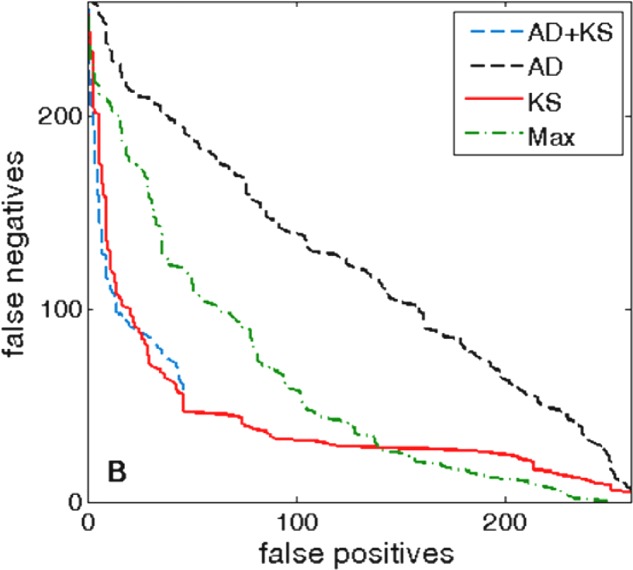
ROC curves, σ^70^ binding. The ROC curves for σ^70^ direct targets, which correspond to different tests, are marked in the legend. The positive and the putative negative sets are the same as in **Figure 3**, and the false negatives and the false positives are estimated with respect to these two sets, where the convergent background distribution is used. In the legend, “Max” corresponds to the standard method for direct target identification (see the first paragraph of this subsection).

Our next goal is to compare the accuracy of KS-based approach, with the standard method for identifying putative targets in bacteria. This method (which we further call “Max”) involves scanning the upstream genomic regions by PSWM, and classifying as putative targets those regions that contain individual binding sites with PSWM scores above certain threshold. To this end, we use the same positives and putative negatives as introduced in the previous subsection, and the null distribution that corresponds to the convergent intergenic regions. In addition, as an alternative to KS test, the AD test can also be used to detect overrepresentation of the binding scores with respect to the null distribution. Consequently, we also address how accurately the two tests (AD and KS) can predict direct targets of σ^70^. The corresponding prediction accuracies are assessed by ROC curves shown in **Figure 4**.

Importantly, we see that KS based approach (the solid red curve) shows a substantially better performance compared to the standard method (the dot-dashed green curve). In particular, note that for the fixed number of false positives there are up to three times fewer false negatives. Such a reduction in the number of false positives is expected, i.e., in accordance with the hypothesis we presented above, since individual sites with high PSWM scores can easily appear by random. On the other hand, their appearance is automatically taken into account through the background distribution, i.e., a potential target will be classified as a hit only if the binding scores in the entire searched region are enriched (overrepresented) with respect to the background distribution.

Furthermore, we see that KS (the solid red curve) leads to a higher detection accuracy compared to AD (the dashed black curve). Moreover, KS test is also much (∼400 times) faster in predicting the direct targets. Consequently, in this application, KS test is both faster and more accurate than AD. Note that this runs opposite to the common paradigm, according to which AD is slower, but more accurate compared to KS (Stephens, 1974).

**FIGURE 5:**
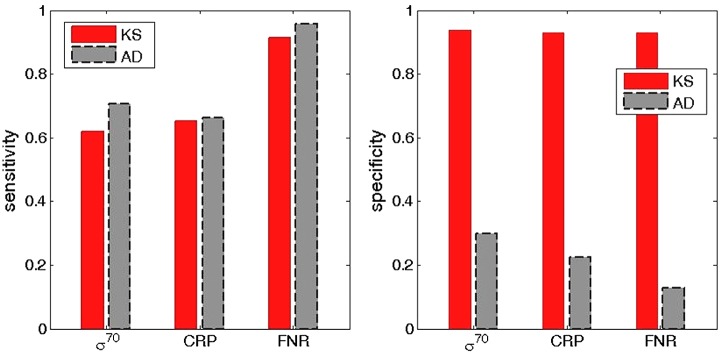
Comparison of sensitivity and specificity for KS and AD methods. The **(left, right)** correspond, respectively, to the sensitivity and specificity estimates, obtained for the usual *P* = 0.05 confidence level. The red and the gray bars correspond to KS and AD methods respectively. The sensitivity and the specificity estimates are shown for σ^70^ (the left bars), CRP (the central bars), and FNR (the right bars). The sensitivity and specificity are calculated as, respectively, TP/P, and TN/N, where TP are true positives, TN true negatives, while P and N are the number of positives and negatives, respectively.

**FIGURE 6:**
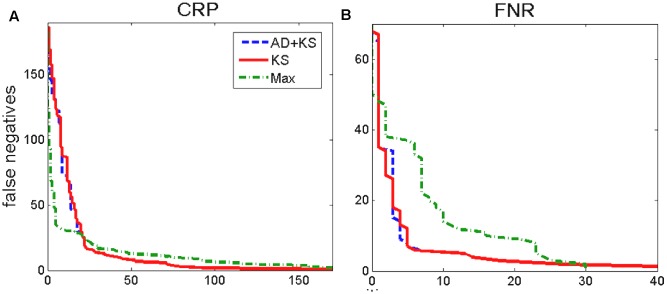
ROC curves, CRP and FNR. The **(A)** and the **(B)** correspond, respectively, to the ROC curves for CRP and FNR. The true positive and the putative negatives are as in **Figure 3**, and the convergent intergenic regions are used for the background distribution. Different tests which correspond to the shown ROC curves are marked in the figure legend.

**FIGURE 7:**
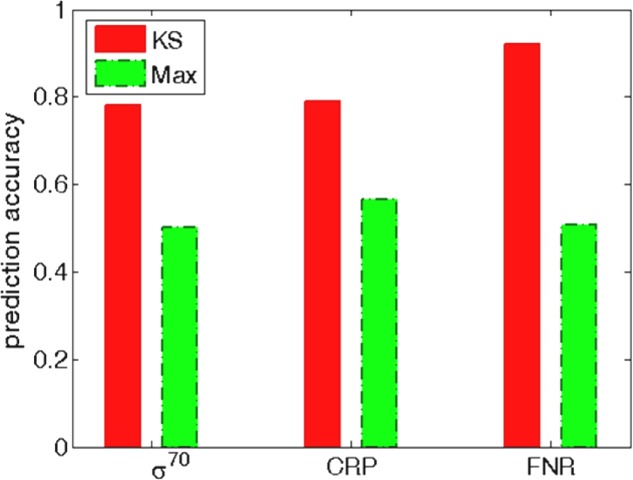
Prediction accuracy for the standard classification thresholds. The threshold in KS search is based on the estimated statistical significance (the usual *P* = 0.05 threshold is taken). The threshold in the PSWM search corresponds to the standard choice where most (98%) of the experimentally determined binding examples would be recovered in the search. The prediction accuracy for these two thresholds is shown for σ^70^ (the left bars), CRP (the central bars), and FNR (the right bars). The prediction accuracy is calculated as (TP + TN)/(TP + FP + FN + TN), where TP (true positives), TN (true negatives), FP (false positives) and FN (false negatives) are calculated for the two methods at the corresponding threshold choices.

To investigate the reason behind the (unexpected) significantly higher accuracy obtained with KS method, in **Figure 5** we compare the sensitivity (the left panel) and the specificity (the right panel) for KS and AD methods. The comparison corresponds to the standard classification threshold (*P* < 0.05) for both methods, and is provided for σ^70^ (analyzed in **Figure 4**) and for CRP and FNR transcription factors (analyzed in **Figures 6**, **7** below). We see that the sensitivity is high, and about the same, for both methods (with AD displaying even slightly larger sensitivity). On the other hand, in the right panel of **Figure 5**, it can be seen that the specificity is much smaller for AD method, which then leads to its lower accuracy compared to KS method. To interpret this result, one should note that we necessarily work with an approximation of the true null distribution, e.g., the negative selection on non-sites in the convergent intergenic regions is likely not the same as in the other intergenic regions, in which the target classification is exhibited. Consequently, the main general advantage of AD method, which is its large sensitivity, becomes a weakness in this application, as small differences with respect to the null distribution (that may also arise from its approximate nature), are (conveniently) not captured by KS, but are classified as statistically highly significant by AD test, leading to low AD specificity (a large number of false positives).

Next, there comes a question if a combination of AD and KS tests can provide an improved accuracy compared to either of the two tests alone. With that respect, we made an algorithm corresponding to a hybrid where the KS test is implemented first to filter-out those upstream regions with clearly insignificant *P*-values, i.e., regions where the actual distribution is clearly too close to the null distribution. Afterward, AD is applied to those regions with distributions that are more different to the null distribution, for which AD performs better. From **Figure 4**, we see that such AD-KS hybrid (the dashed blue curve) indeed shows a substantially better accuracy compared to AD test alone, and has a similar accuracy to KS test alone. This result is consistent with the discussion above, i.e., when AD test alone is used, a number of the upstream regions that are eliminated by KS test, are falsely classified as targets by AD (since, due to small specificity, AD proclaims even small difference between the distributions as being significant). On the other hand, when in AD-KS hybrid AD is applied only to those distributions that are more different with respect to the null distribution (therefore bypassing its main problem of low specificity), the accuracy becomes similar to KS test. As an outlook, note that AD-KS hybrid might be further improved by optimizing the threshold for KS selection. We will further concentrate only on KS and AD-KS tests, as they have a much better accuracy to AD test alone.

We next come back to assessing KS approach for two more standard binding score distributions (see **Figure 1**), exhibited by CRP and FNR transcription regulators. We here construct the positive and the putative negative sets in the same way as for σ^70^, i.e., the positives correspond to the intergenic regions where the transcription regulator binding is experimentally shown, while the putative negatives correspond to the sequences deep inside the coding regions, where functional binding is not expected. The corresponding ROC curves are shown in **Figure 6**.

For FNR (**Figure 6B**), we obtain similar results as for σ^70^, i.e., KS (and KS-AD hybrid) lead to a significantly better ROC curve performance compared to the standard method (e.g., for a fixed false negative number, there is a several times smaller number of false positives for KS). On the other hand, we see that for CRP the two curves (KS and the standard method) have apparently similar performances, i.e., while the standard method shows better performance at low false positive numbers, it is outperformed by KS at higher false positives. The similar performance of KS in the case of CRP is not surprising, i.e., is likely a consequence of the fact that functional binding dominates over non-sites in this case, as implied by the large PSWM score overrepresentation exhibited in such case (see the upper left panel in **Figure 1**). Consequently, the results in **Figure 5** are in line with our main hypothesis that the main utility of KS approach is in accurate classification of non-sites.

### Statistical Significance and the Classification Threshold

Independently from the ROC performance, KS has a significant advantage of straightforwardly assigning statistical significance to each predicted target, which is normally not available for standard PSWM search (see Introduction). We here explore the utility of such robust statistical significance estimate with the example of assigning a classification threshold. With the KS approach a natural threshold choice is provided by the *P*-value, typically set to *P* = 0.05. As such a natural choice is normally not available for standard PSWM search, the threshold is usually set so that almost all (∼98%) of the experimentally determined binding sites from which PSWM is constructed are recovered in the search. In **Figure 7**, we explore the search accuracy associated with the two choices of the binding threshold, i.e., *P* = 0.05 for KS method and the standard threshold (see above) for PSWM search.

We see that the threshold based on KS significance estimate leads to much higher prediction accuracy for σ^70^ and FNR, which is expected based on the significantly better ROC performance of KS in these two cases (**Figures 4**, **6B**). Moreover, in **Figure 7** we also see notably higher prediction accuracy in the case of CRP, where a similar ROC performance was observed for KS and standard PSWM search (**Figure 6A**). Consequently, the notably higher search accuracy for KS in the case of CRP observed in **Figure 7** is based on the more optimal choice of the classification threshold. This underlines the advantage of the threshold choice based on the robust statistical significance measure.

## Conclusion and Outlook

We here proposed a new computational approach to direct regulatory target prediction. The approach is based on assessing the significance of the difference between PSWM scoring distributions, which correspond to the upstream genomic regions, and the background distribution. As a consequence, P-value is assigned to the entire upstream region, instead of searching for individual high-scoring binding sites. We implemented this approach through classical Kolmogorov–Smirnov and Anderson–Darling tests, as well as through a hybrid of these two approaches. Surprisingly, and contrary to the current paradigm, we have seen that the approach based on Kolmogorov–Smirnov test leads to a higher search accuracy compared to Anderson–Darling based approach, while also being (as expected) computationally less demanding. While the hybrid approach has a substantially higher accuracy compared to Anderson–Darling test, it does not outperform the simpler Kolmogorov–Smirnov test. We interpreted this result by Anderson–Darling test classifying small differences with respect to the background distribution as true binding targets, leading to low specificity of the approach.

We furthermore showed that the Kolmogorov–Smirnov based approach leads to a substantially higher accuracy compared to the standard approach, reducing the number of false positives for several times. Moreover, a clear advantage of Kolmogorov–Smirnov approach is that it straightforwardly assigns statistical significance to any tested upstream intergenic region. We demonstrated this advantage on the example of the classification threshold, where we have seen that the robust significance estimate provided by Kolmogorov–Smirnov leads to a much more optimal threshold choice. We find that genomic regions, where functional binding is not expected, provide better background compared to randomized genomic regions. We here, i.e., for analysis of prokaryotic transcription regulation, used convergent intergenic regions for background distribution. In eukaryotes the choice of background distribution would be more complicated and remains to be investigated, where one possibility would be to take genomic sequences far from coding regions (where there may not be much TFBS).

To prove this new concept in the direct regulatory target prediction, we tested it in the case of pleiotropic bacterial regulators. This allowed a more straightforward interpretation of the obtained results, while testing the method on some of the classical problems otherwise characterized by low prediction specificity. As an outlook, the method proposed here is of a general significance, and it will be in the future also implemented in the more complicated case of direct target prediction for eukaryotic transcriptional regulators. Moreover, while the model was here applied in the context of PSWM, more complex models which take into account interdependences of nucleotides in TFBS were also developed (Eggeling et al., 2015; Kulakovskiy et al., 2016; Nettling et al., 2017). While these methods lead to a better performance in some cases, more often (simpler) PSWMs perform better, which is likely due to overfitting, i.e., due to a limited number of TFBS from which the model is trained (Benos et al., 2002; Nguyen and Androulakis, 2009). Therefore, despite the limitations of PSWMs, they are still the leading approach in TFBS search (Nguyen and Androulakis, 2009; Fazius et al., 2011). In any case, the new approach proposed here does not depend on the scoring method (i.e., if a classical PSWM, or a higher order model, is used), since the approach is based on comparing the distributions of the scores (i.e., is not limited by how the actual scores are calculated). Consequently, the KS approach proposed here might present a general method of choice for efficiently and accurately predicting target loci of transcription regulators.

## Materials and Methods

### Defining the Upstream Genomic Regions

The *E. coli* intergenic sequences are divided in two groups, where binding of transcription regulators is expected (other intergenic regions) and not expected (convergent intergenic regions). The other intergenic regions, and the convergent intergenic regions, include, respectively, those that are located upstream of at least one adjacent gene, and downstream of both of the adjacent genes.

For the positive set in σ^70^ case, in KS, AD and KS-AD hybrid searches, we take those *E. coli* intergenic sequences that contain σ^70^ binding sites with experimental evidence from RegulonDB database (Gama-Castro et al., 2011), which results in the total of 263 upstream genomic regions. Similarly, for the positive set in CRP and FNR case, we take these intergenic sequences with experimental evidence of the regulator binding from RegulonDB database. For the putative negative set, we use the same number of sequences, with the same length, as those in the positive set, but now sampled from ORF (coding sequences), where we exclude 50 bps at both 5′ and 3′ ends; this is done to exclude the flanking sequences, in which σ^70^ binding sites are sometimes located.

For obtaining the randomized distribution, an ensemble of randomized sequences was constructed, by sampling all trinucleotide probabilities in the intergenic regions. The randomized sequences were searched, and the corresponding randomized scoring distributions are obtained, in the same manner as for the upstream genomic regions, which is further described below.

### PSWM Scoring Distributions

CRP and FNR PSWM were constructed from the binding sites assembled in DPInteract database (Robison et al., 1998), through the standard information-theory based procedure (Stormo, 2000). σ^70^ PSWM were constructed starting from recent *de novo* alignment (Djordjevic, 2011), where the promoter elements were systematically aligned starting directly from the experimentally determined TSS. Briefly, the alignment includes: -10 and -35 elements, spacer weights corresponding to variable spacer length (between 15 and 19 bps), conserved sequences upstream of -10 element. PSWMs for CRP, FNR and σ^70^ search are provided in Supplementary Table 1. Scores were assigned to each DNA segment in the upstream genomic and the randomized sequences using these PSWM, from which the corresponding scoring distributions were generated.

### KS, AD, and KS-AD Hybrid Based Searches

For KS based search, one-sided Kolmogorov-Smirnov test was used. For each tested upstream intergenic region PSWM score distribution was generated as described above, and compared with an appropriate background distribution whose CDF was constructed. This comparison results in *P*-value and *D* score for each tested upstream genomic segment. The threshold on *D* scores was then moved in order to change the number of false positives and false negatives, and construct the ROC curves.

For AD based search, the MATLAB based routine ‘adtest’ was used, where PSWM score distributions corresponding to upstream genomic region, and the background distribution were compared. For each tested upstream genomic region, *P*-value and AD test statistics (‘adstat’) was sampled. The ROC curves were constructed based on ‘adstat’ scores.

For KS-AD hybrid search, KS and AD tests were implemented as described above, with KS test used first to exclude the upstream intergenic regions with low difference between the two distributions. A liberal *P*-value threshold of 0.5 was used in this exclusion, so that only the upstream genomic regions with very low significance are eliminated by KS test. The rest of the upstream regions are then subjected to AD test, which is used to calculate *P*-value and AD test statistics. The codes for KS, AD and KS-AD hybrid approaches are available upon request.

## Author Contributions

All authors have given approval to the final version of the manuscript. MarD conceived the work, with the help of MagD and EZ. MarD and MagD implemented the method and performed the analysis. All the authors interpreted the results. MarD wrote the paper, with the help of MagD and EZ.

## Conflict of Interest Statement

The authors declare that the research was conducted in the absence of any commercial or financial relationships that could be construed as a potential conflict of interest.
