# Supplementary material for: Scoring Targets of Transcription in Bacteria Rather than Focusing on Individual Binding Sites
Source: Front Microbiol. 2017 Nov 22;8:2314. doi: 10.3389/fmicb.2017.02314 (PMC5702782; doi:10.3389/fmicb.2017.02314)
Supplement: Supplementary file 1 [file Table_1.PDF]

Weight matrix for CRP search

Columns 1 through 6

A	0	0	0	-4.9285	-3.1444	-3.4457
T	-0.0898	-0.2417	0	0	-2.1324	0
C	-2.6762	-2.0760	-1.1910	-1.2886	-3.4989	-1.3137
G	-1.4327	-1.0445	-0.4089	-2.5990	0	-4.9537

Columns 7 through 12

A	-2.5401	0	-1.3518	-0.8937	-0.7815	-0.3952
T	-2.5401	-5.0255	0	-0.5355	0	-0.7914
C	-5.2527	-2.3235	-0.5897	0	-0.0113	-0.8696
G	0	-2.3235	-0.6927	-0.5219	-1.0820	0

Columns 13 through 18

A	-1.0003	0	-1.6670	-1.9596	0	-1.4181
T	-1.0003	-0.0634	0	-2.7561	-2.3743	-1.7630
C	-0.9487	-0.0967	-2.0608	0	-3.0919	0
G	0	-0.5377	-1.2526	-5.2030	-0.9633	-5.0064

Columns 19 through 22

A	0	-0.5496	-0.5046	-0.5307
T	-1.6670	0	0	0
C	-1.7900	-0.5608	-2.9391	-0.9373
G	-1.4018	-0.8055	-4.6683	-1.4748

Weight matrix for FNR search

Columns 1 through 6

A	-0.5259	0	0	0	-2.3364	-3.9169
T	-0.5259	-1.2883	-1.2343	-0.1477	0	0
C	0	-1.0426	-3.6811	-1.5142	-3.8443	-3.9169
G	0	-1.0426	-3.6811	-3.2434	-3.8443	-3.9169

Columns 7 through 12

A	-4.1153	0	-3.8443	-0.6489	0	0
T	-2.6074	-1.6823	0	0	-0.3210	-3.4860
C	-4.1153	-3.7660	-2.1150	-0.1230	-1.5142	-0.3004
G	0	-3.7660	-3.8443	-1.3665	-1.5142	-3.4860

Columns 13 through 18

A	-0.6483	0	-1.0392	-4.1883	0	0
T	-1.8531	-3.3721	0	-4.1883	-3.6811	-3.7660
C	0	-0.6701	-1.7568	0	-3.6811	-3.7660
G	-0.6590	-0.6701	-1.7568	-4.1883	-0.9791	-1.4365

Columns 19 through 22

A	-0.5308	0	0	-0.5308
T	0	-0.6594	-0.1733	0
C	-0.9139	-1.0426	-3.0957	-1.5142
G	-1.5142	-3.3721	-0.3938	-0.9139

Weight matrices for σ70 search, according to the alignment provided in (Djordjevic, 2011)

Weight matrix -35 element:

	-35	-34	-33	-32	-31	-30
A	-0.93	-2.10	-1.19	0	-0.34	0
T	0	0	-1.44	-0.51	-1.31	-0.29
C	-1.52	-1.14	-4.44	-0.16	0	-0.79
G	-1.93	-3.19	0	-0.65	-1.68	-0.32

Weight matrix -15 element:

	-15	-14	-13	-12
A	-0.51	-1.02	-0.84	-3.39
T	0	-0.75	-0.29	0
C	-0.04	-0.73	-0.27	-1.42
G	-0.16	0	0	-2.09

Weight matrix short -10 element:

	-11	-10	-9	-8	-7
A	0	-0.40	0	0	-3.45
T	-2.36	0	-1.27	-1.65	0
C	-4.23	-1.02	-1.26	-0.65	-2.69
G	-4.60	-0.90	-1.63	-1.53	-3.97
